# Anthracycline-Induced Cardiotoxicity in Breast Cancer Patients from Southern Sri Lanka: An Echocardiographic Analysis

**DOI:** 10.1155/2020/1847159

**Published:** 2020-11-12

**Authors:** Jayasinghe Arachchige Nirosha Sandamali, Ruwani Punyakanthi Hewawasam, Madappuli Arachchige Chaminda Sri Sampath Fernando, Kamani Ayoma Perera Wijewardana Jayatilaka, Ranji Duleep Madurawe, Periyasamy Pillai Sathananthan, Upul Ekanayake, Jayamini Horadugoda

**Affiliations:** ^1^Department of Medical Laboratory Science, Faculty of Allied Health Sciences, University of Ruhuna, Galle, Sri Lanka; ^2^Department of Biochemistry, Faculty of Medicine, University of Ruhuna, Galle, Sri Lanka; ^3^Department of Economics & Statistics, Faculty of Social Sciences & Languages, Sabaragamuwa University of Sri Lanka, Belihuloya, Sri Lanka; ^4^Teaching Hospital, Karapitiya, Sri Lanka

## Abstract

Anthracycline-induced cardiotoxicity has never been investigated in Sri Lanka. Therefore, this study was conducted to determine the prevalence of anthracycline-induced cardiotoxicity in breast cancer patients using echocardiographic findings. A prospective cohort study was performed. All newly diagnosed breast cancer patients who were administered with anthracycline and cyclophosphamide (AC schedule) for the first time were enrolled in the study. In the hospital setting, anthracycline is administered only as a combination therapy, and only this combination was selected to limit the effect of other cardiotoxic chemotherapy agents. Records of echocardiography were obtained: one day before anthracycline chemotherapy (baseline), one day after the first chemotherapy dose, one day after the last chemotherapy dose, and six months after the completion of anthracycline chemotherapy. Following parameters were recorded from the echocardiography results: ejection fraction (EF, %), fractioning shortening (FS, %), posterior wall thickness, left ventricle (PWT, mm), the thickness of interventricular septum (IVS, mm), left ventricular end-diastolic diameter (LVEDD, mm), and left ventricular end-systolic diameter (LVESD, mm). Statistical analysis of the echocardiography results was performed using ANOVA at four stages. A *p* value <0.05 was considered significant. Subclinical cardiac dysfunction was defined as a fall of EF >10% during the follow-up echocardiography. There was no significant change (*p* > 0.05) between the baseline echocardiographic parameters and one day after the 1^st^ anthracycline dose. However, significant differences (*p* < 0.05) were observed between the baseline echocardiographic parameters and one day after the last anthracycline dose and six months after the completion of anthracycline therapy with a gradual and progressive deterioration in functional parameters including EF, FS, PWT, and IVS over time. There were 65 patients out of 196 (33.16%) who developed subclinical cardiac dysfunction six months after the completion of anthracycline chemotherapy. The prevalence of subclinical anthracycline-induced cardiotoxicity was relatively higher in these patients. An equation was also developed based on left ventricular ejection fraction (LVEF) to predict the anthracycline-induced cardiotoxicity of a patient six months after the completion of anthracycline chemotherapy. We believe that this will help in the monitoring of patients who undergo anthracycline therapy for cardiotoxicity. It is recommended to carry out a long-term follow-up to detect early-onset chronic progressive cardiotoxicity in all patients who were treated with anthracycline therapy.

## 1. Introduction

The number of cancer survivors has increased dramatically over the past few decades with the advancement of new cancer treatment regimes. In the USA, 16.9 million cancer survivors were expected by Jan 2019, and it is projected to be increased to 21.7 million by the year 2029 [1]. However, they are vulnerable to a plethora of conditions resulting primarily from the effects of chemotherapy, and anthracycline-induced cardiac dysfunction has been recognized as a serious side effect since its introduction in the 1960s [2].

Anthracyclines are a group of antibiotics that made remarkable advances in the treatment of a wide variety of solid organ tumors and hematologic malignancies, including leukemia, lymphoma, breast cancer, lung cancer, multiple myeloma, and sarcoma [3]. Among all anthracyclines, doxorubicin is the most commonly used anticancer drug [4]. However, the clinical utility of doxorubicin is hampered due to some side effects such as hematopoietic suppression, nausea, vomiting, extravasation, alopecia, and cardiotoxicity [5]. Several studies have shown that cumulative dose-dependent cardiotoxicity is the major limitation of doxorubicin usage [6]. The main mechanism of action of doxorubicin in cancer cells involves the intercalation into deoxyribonucleic acid (DNA) and the disruption of topoisomerase-II-mediated DNA repair [7]. Although the mechanism of doxorubicin-induced cardiotoxicity is multifactorial, free radical-induced oxidative stress, mitochondrial dysfunction, and calcium overload are considered as major contributory factors [3].

The heart is more vulnerable than other tissues due to low levels of antioxidant enzymes such as peroxidase, catalase, and superoxide dismutase [8]. Mitochondria have been identified as the main subcellular organelles injured in the heart as cardiolipin in mitochondrial inner membrane, which is required for the proper functioning of the electron-transport chain proteins, forms a nearly irreversible complex with doxorubicin cationic drug [8, 9].

Doxorubicin-induced cardiotoxicity may range from asymptomatic electrocardiographic (ECG) changes to decompensated cardiomyopathy which is characterized by decreased left ventricular ejection fraction (LVEF) [8]. Prevalence of acute and subacute cardiotoxicity is about 20-30%, and it is usually evident with transient ECG changes including nonspecific ST- and T-wave flattening, decreased QRS voltage, and prolongation of QTc interval, while chronic cardiotoxicity is associated with an irreversible nonischemic dilated cardiomyopathy [10]. Cardiomyopathy is more common in patients receiving a high cumulative dose of doxorubicin; extremes of age; female gender; patients with underlying cardiovascular diseases; concomitant treatment with cyclophosphamide, trastuzumab, or paclitaxel; and in patients who undergo prior mediastinal radiation therapy [11, 12].

Reduction of ejection fraction (EF) provides advanced warning of cardiotoxicity in patients receiving anthracycline chemotherapy even prior to clinical signs of left ventricular dysfunction appear. Transthoracic echocardiography is a reliable, noninvasive, and sensitive method for the detection of subclinical cardiac dysfunction [13]. According to literature, very few studies have been carried out in Asian countries regarding the prevalence of anthracycline-induced cardiotoxicity. However, studies have never been carried out in Sri Lanka on anthracycline-induced cardiotoxicity. Therefore, the current study was conducted to determine the prevalence of anthracycline-induced acute and early-onset chronic progressive cardiotoxicity in cancer patients admitted to the oncology unit, Teaching Hospital, Karapitiya, Sri Lanka, using an echocardiographic analysis.

## 2. Materials and Methods

Ethical approval for the study was obtained from the Ethical Review Committee, Faculty of Medicine, University of Ruhuna, Sri Lanka (23.10.2014:3.10). A prospective cohort study was performed from November 2015 to February 2019 at the Teaching Hospital, Karapitiya, Sri Lanka. All newly diagnosed breast cancer patients who were administered with anthracycline and cyclophosphamide (AC) chemotherapy for the first time and who gave informed consent were enrolled in the study. In the hospital setting, anthracycline is administered only as a combination therapy, and only this combination was selected to limit the effect of other cardiotoxic chemotherapy agents. Any patient who was in a critical state or end stage of cancer and who were unable to give informed consent; patients who received chemotherapy that does not belong to the anthracycline family; patients who were diagnosed with any structural heart disease, hypertension, or cardiomyopathy; and patients who developed any acute noncardiac complications before the recruitment to the study were excluded from the study. Once the consent was given, an interviewer-administered questionnaire was used to collect the sociodemographic data and other relevant information related to the study. Any other information required for the study was also collected by the investigator from the bed head ticket (BHT) and investigation reports where appropriate. Anthropometric measurements were also obtained by the principal investigator. Physiological measurements such as baseline heart rate, systolic/diastolic blood pressure, were measured by a digital blood pressure apparatus. History of chronic conditions and drug usage was also noted. The positivity or negativity of estrogen receptor (ER), progesterone receptor (PR), and human epidermal growth factor receptor 2 (Her-2) was also recorded from the reports available during the study. The type and stage of malignancy were recorded. The type of anthracycline drugs used, route of administration, highest dosage, cumulative dosage, and the number of cycles of anthracyclines administered were noted. Details of radiotherapy were also collected. Records of echocardiography done by the cardiologist were obtained from each patient on the following occasions: one day before chemotherapy, one day after the first chemotherapy dose, one day after the last chemotherapy dose, and six months after the completion of chemotherapy. Following parameters were recorded from the echocardiography results; ejection fraction (EF, %), fractioning shortening (FS, %), left ventricular posterior wall thickness (PWT, mm), the thickness of interventricular septum (IVS, mm), left ventricular end-diastolic diameter (LVEDD, mm), and left ventricular end-systolic diameter (LVESD, mm).

### 2.1. Statistical Analysis

The statistical analysis was performed using the MINITAB 18 software package. Continuous variables such as age, weight, height, BMI, cumulative anthracycline dose, and radiation were presented as mean ± SD. Statistical analysis of echocardiography results was analyzed using paired *t*-test at four stages. The level of significance was 5%. Subclinical cardiac dysfunction was defined as a fall of EF >10% from the baseline EF value during follow-up echocardiography. There are ten categorical variables (cancer level, treatment type, other drugs, cancer relatives, marital state, diabetes mellitus, dyslipidemia, ER, PR, and HER2 receptor state) and six continuous variables (age, weight, height, BMI, cumulative dose, and radiation) that can be taken as potential explanatory variables to explain the behavior of percentage change in six echocardiography variables (EF, FS, PWT, IVS, EDD, and ESD) after six months of completion of anthracycline chemotherapy. A simple linear regression model was created to predict EF value after the six months of completion of anthracycline chemotherapy for the unobserved future observations. Point estimation and interval estimation were also found to estimate 95% confidence interval for the future prediction model. Binary logistic regression analysis was carried out to investigate the relationship between the cardiac risk factors and the occurrence of subclinical cardiotoxicity. The odds ratios (ORs) and 95% confidence intervals (CIs) were estimated from regression coefficients.

## 3. Results

Patients selected for the study (196) had no clinical signs and symptoms of cardiac dysfunction during the study period (November 2015 to March 2019). All patients were females with breast cancer as there were limitations to select patients who receive anthracycline drug regimes in the actual clinical setup.

The demographic profile and other clinical characteristics of the patients enrolled in the study (age, weight, height, BMI, cumulative dose, radiation, systolic BP, and diastolic BP) were presented in the form of mean ± standard deviation in Table 1, and cumulative dose of anthracycline and radiation therapy varied more than the other factors considered. Other baseline characteristics of the patients enrolled in the study including Nottingham grade of cancer; type of anthracycline; number of anthracycline cycles; second-line drugs; and blood relatives with cancer, marital state, diabetes, dyslipidemia, ER, PR, and Her 2 receptor status were presented as percentage frequencies in Table 2.

Echocardiographic comparison of baseline myocardial function one day after the first anthracycline dose, one day after the last anthracycline dose, and six months after completion of anthracycline therapy is shown in Table 3. There was a gradual and progressive deterioration in functional parameters of echocardiography including EF, FS, PWT, and IVS over time in patients who received anthracycline chemotherapy. Although these patients did not develop apparent cardiac dysfunction, they are at a higher risk of developing cardiac dysfunction in the future. In this study, subclinical cardiac dysfunction was identified as a fall of EF >10% from the baseline EF value. Although EF value was reduced in some patients one day after the last dose of anthracyclines, none of them had a >10% reduction suggesting that no one developed subclinical cardiac dysfunction. However, there were 65 patients out of 196 (33.16%) who had a reduction of EF value >10% from the baseline results six months after the completion of anthracycline chemotherapy.

When analyzed the percentage variability explained by the demographic and clinical features including age, weight, height, BMI, cumulative dose, radiation, cancer level, treatment type, other drugs, cancer relatives, marital state, diabetes, dyslipidemia, ER, PR, and Her 2 status against the echocardiography parameters six months after the completion of anthracycline chemotherapy, the highest variability was observed for the EF among the six echocardiography parameters. Therefore, EF was used for further analysis, and the correlation between the EF and the demographic and clinical characteristics including age, weight, height, BMI, cumulative dose, and radiation were determined and presented in Table 4. A statistically significant (*p* < 0.05) linear relationship was observed with five variables except the cumulative dose of anthracyclines. *R*^2^ of the simple linear regression models of echocardiography measurements six months after the last anthracycline chemotherapy dose against a day after the last anthracycline chemotherapy dose are given in the second column of Table 5. The *R*^2^ of the multiple linear regression models of demographic and other patient characteristics are given in the last column. In this study, it was unable to observe any sizable contribution from the above 16 characteristics in the presence of the echocardiography measurements a day after the last treatment. Therefore, it can be suggested that the simple linear regression model can be used to describe the behavior of percentage change of the echocardiography variables six months after the last anthracycline chemotherapy dose against the measurement a day after the last anthracycline chemotherapy dose. According to the results, it is obvious that the percentage change of some echocardiography parameters six months after the completion of anthracycline chemotherapy correlates with the echocardiography parameters one day after the last anthracycline chemotherapy dose. The model fitted for EF was the best as it explains 89% of the total variation. Therefore, it is suggested that EF value six months after the completion of anthracycline chemotherapy can be explained by the EF observed on the day after the last anthracycline chemotherapy dose. The predicted *R*^2^ and *R*^2^ values for the above-fitted model were 88.7% and 88.9%, respectively. The higher *R*^2^ values indicate the high capability of this model in predicting EF value six months after the completion of anthracycline chemotherapy.

A point estimate for the unobserved predicted EF value six months after the completion of anthracycline chemotherapy (EF_6_) given that the observed value of EF difference the day after the last anthracycline chemotherapy dose from the baseline EF value (EF_*D*_) was calculated as,

(1) $$\begin{array}{c} {\text{EF}}_{6}=2.002+1.2332{\text{EF}}_{D}. \end{array}$$

Table 6 indicates the association of risk factors with the occurrence of subclinical cardiotoxicity. According to the binary regression analysis, BMI was the most independently associated (*p* < 0.05) risk factor in these patients. Overweight patients had an OR of 4.02 and obese patients had an OR of 4.21 compared to the patients having a healthy weight. The second significantly associated (*p* < 0.05) risk factor for the occurrence of subclinical cardiotoxicity was the treatment with chest wall radiation which showed an OR of 1.33. Although other cardiac risk factors including age >60 years (OR; 1.14), treatment with doxorubicin (OR; 1.80), dyslipidemia (OR; 1.28), and trastuzumab administration (OR; 2.19) also had an independent association with the occurrence of subclinical cardiotoxicity with OR >1, they were not significant.

## 4. Discussion

Dose-dependent cardiotoxicity of anthracyclines is considered the most serious complication of anthracycline therapy, and it has negative impacts on the cardiac outcome of cancer patients and a major limitation on their therapeutic potential [14]. Cardiotoxicity evident in anthracycline chemotherapy is observed as acute cardiotoxicity which usually occurs during or immediately after infusion of anthracyclines, early-onset chronic progressive cardiotoxicity which occurs within one year of discontinuation of chemotherapy, and late-onset chronic progressive cardiotoxicity which occurs after one year of exposure [12]. The incidence of acute cardiotoxicity is about 3-21%, while late cardiotoxicity is about 0-57% [15]. As the subclinical cardiotoxicity is more prevalent in patients on anthracycline chemotherapy, early detection may increase the survival rate of patients [16]. Therefore, in this study, echocardiography was performed at four stages and LVEF measurement was used to assess the cardiac systolic functions as it is the most accepted indicator of the prognosis.

According to literature, there are many definitions for cardiotoxicity based on the changes in LVEF [4, 16, 17]. Based on the previous findings, the American and European Society of Echocardiography Expert Consensus defined cardiotoxicity related to cancer therapeutics as a reduction of LVEF >10%, to a value below 53% [18]. Therefore, in the current study, LVEF <53% with symptoms was considered as clinical cardiotoxicity and a reduction of LVEF >10% was considered as subclinical cardiotoxicity. According to the results of the current study, some patients showed a reduction of LVEF measurements one day after the last dose of anthracycline chemotherapy, but no one had a reduction of up to 10%. Although 33.16% of patients had >10% reduction in LVEF measurements six months after the cessation of anthracycline chemotherapy, none of them had any overt clinical signs or symptoms of cardiomyopathy. This may be due to the method of anthracycline administration. In the Sri Lankan hospital setup, anthracycline is administered as four divided doses every three weeks or four divided doses administered in three consecutive days every three weeks. According to a clinical study done by Valdivieso et al., endomyocardial biopsies of patients who received doxorubicin chemotherapy revealed that a bolus dose given in every three weeks cause significantly higher damage than three divided doses given every week or three divided doses given in three consecutive days in every three weeks [19]. It was also reported that patients who received divided doses can tolerate a higher cumulative dose of doxorubicin, with no significant difference in tumor response rates and overall survival rates among the groups. The weekly schedule also offered a longer duration of response than the bolus dose.

Early-onset chronic progressive cardiotoxicity is evident within one year after the cessation of chemotherapy, and this form of cardiotoxicity has been identified as the most relevant type of cardiotoxicity in the clinical setup [14]. Therefore, in the present study, echocardiography measurements were obtained six months after the completion of chemotherapy, and these results are in line with the study done by Khattryet et al. for adult Indian patients who underwent anthracycline chemotherapy and showed a subclinical cardiac dysfunction up to 27% [20]. Another study conducted by Shaikh et al. for children in Pakistan showed that 25% of patients developed cardiac dysfunction one year after the completion of anthracycline chemotherapy [15]. In the present study, an equation was developed based on LVEF to predict the anthracycline-induced cardiotoxicity of a patient six months after the completion of anthracycline chemotherapy using the difference in the LVEF measurement that was taken one day after the last dose of anthracycline chemotherapy from the baseline value. It will help to predict the EF value after six months and to monitor and treat the patients before developing cardiotoxicity symptoms.

It is believed that anthracyclines cause an enlargement of left ventricles which increase both systolic and diastolic diameter with a decreased value in EF and FS [15]. In the present study, FS was significantly reduced (*p* < 0.001) after the last dose of anthracycline and six months after the completion of anthracycline chemotherapy. These results are consistent with previous studies done by Shaikh et al. and Lipshultz et al. [15, 21]. EF can provide advanced warning of anthracycline-induced cardiotoxicity even before the clinical signs and symptoms appear [22]. In this study, average EF at the baseline after the last anthracycline dose and six months after the completion of anthracycline was 68.77%, 65.58%, and 63.46%, respectively, and there was a significant difference (*p* < 0.001) between baseline values and the other two stages. A study conducted in Uganda for adult cancer patients who received anthracycline chemotherapy showed 66.4% of EF at the baseline and 63.9% three months after the completion of anthracycline chemotherapy which showed a significant difference (*p* < 0.05) against the baseline results [23]. These results were compatible with the results obtained in the present study. Many previous studies performed using echocardiography have shown a significant reduction of LVEF compared to the baseline values [12, 21, 22]. It is reported that anthracycline-induced cardiotoxicity reduces IVS and PWT of left ventricles which leads to myocardial dysfunction [13]. In this study, a significant reduction (*p* < 0.001) was shown in PWT after the last dose of anthracycline chemotherapy and six months after the completion of the treatment. IVS was also reduced significantly after the last dose (*p* < 0.05) and six months after the completion of anthracycline therapy (*p* < 0.001). Several other studies also have found significant differences in PWT and IVS between the preanthracycline and the postanthracycline treatment [15, 16, 22].

Previous studies have identified some risk factors for the anthracycline-induced cardiotoxicity. Some of them are high cumulative doses of anthracycline, female gender, age >65years, hypertension, preexisting cardiac diseases, mediastinal/chest wall radiation, treatment with cyclophosphamide, taxel, and trastuzumab [11]. In the present study, we excluded the patients with previous heart diseases and hypertension. EF value was selected to assess the risk as it is the most important echocardiography parameter that correlates with symptoms, prognosis, events, and complications in many cases of left ventricular dysfunctions [13, 15].

In our clinical setup, anthracycline is administered in combination with cyclophosphamide or several other drug agents. As we selected only the patients receiving anthracycline and cyclophosphamide (AC schedule), the prevalence of this risk factor was 100%. This is one of the limitations of the present study. According to some previous case reports, it is obvious that cyclophosphamide is also a cardiotoxic agent which can induce a different degree of cardiomyopathy [24, 25]. Some studies have also shown that the incidence of acute heart failure may be between 7 and 33% in patients receiving more than 150 mg/kg of cyclophosphamide [26, 27]. However, according to literature, the actual incidence of cyclophosphamide-induced cardiotoxicity is difficult to be established as different doses of cyclophosphamide have been used in different case studies, and this drug is often administered with other cardiotoxic drugs [24]. In the present study, it can be assumed that the administration of cyclophosphamide simultaneously may have an effect to induce subclinical cardiotoxicity in patients receiving anthracycline chemotherapy. According to the relative risk analysis of the patients in the present study, BMI was a major independent risk factor for the development of subclinical cardiotoxicity. According to the literature, it was proven that obese breast cancer patients treated with anthracyclines have a higher risk for the reduction of LVEF [28]. Also, chest wall irradiation therapy had significant odds (OR = 1.36, *p* = 0.006) for the occurrence of subclinical cardiotoxicity. Previous studies also suggested that mediastinal irradiation may develop severe cardiovascular complications in 1-2.2% of breast cancer patients [29, 30]. According to the risk analysis, factors including age, treatment with doxorubicin, dyslipidemia, and administration of trastuzumab had odds of more than 1. Therefore, all these factors showed higher odds for the occurrence of subclinical cardiotoxicity in breast cancer patients of the present study. The incidence of trastuzumab-related cardiac failure is about 2-7% which increases with age >50 years and prior treatment with anthracyclines [29]. In the present study, the prevalence of subclinical cardiac dysfunction was higher (33%) compared to other Asian studies [15, 20]. The reason for this may be due to a higher rate of risk factors that cannot be excluded from this study. However, acute or overt clinical symptoms could not be seen in these patients. It may be due to the infusion method of chemotherapy which may lower the cardiotoxic effects [17]. In our clinical setup, anthracyclines and taxel are given in divided doses, and the infusion rate is very slow (it takes at least three hours for the infusion).

There were several other limitations of this study that need to be mentioned. This study was conducted in a single center; therefore, data are more related to the Southern province of Sri Lanka. Only the female patients with breast cancer could be selected for the study to maintain consistency and to reduce the interference from multiple other factors related to anthracycline-induced cardiotoxicity. During the six months follow-up period, patients were given second-line treatment as well. They may also have been exposed to mediastinal irradiation. Therefore, the final echocardiography data obtained may be contaminated by the administration of nonanthracycline drugs and their toxicities and by radiation therapy.

## 5. Conclusion

The prevalence of subclinical anthracycline-induced cardiotoxicity was relatively higher in these patients. An equation was developed based on LVEF to predict the anthracycline-induced cardiotoxicity of a patient six months after the completion of anthracycline chemotherapy using the LVEF measurement that was taken one day after the last dose of anthracycline chemotherapy. This will help in the monitoring of patients after they were treated with anthracycline chemotherapy. Finally, it is recommended to carry out long-term follow-up to detect early-onset chronic progressive cardiotoxicity in all patients who were treated with anthracycline therapy to increase their quality of life as cancer survivors.

## Figures and Tables

**Table 1:** Demographic and clinical characteristics of patients who received anthracycline chemotherapy.

Demographic and clinical characteristics of patients	Mean (SD)
Age (years)	53.6 (11.0)
Weight (kg)	53.5 (10.3)
Height (m)	1.5 (0.1)
BMI (kg/m^2^)	24.2 (4.3)
Cumulative dose (mg)	340.8 (37.0)
Radiation received (Gy)	49.4 (22.9)
Systolic BP (mmHg)	115.3 (8.1)
Diastolic BP (mmHg)	74.9 (5.8)

BMI: body mass index; BP: blood pressure.

**Table 2:** Percentage frequency of the other baseline characteristics of patients who received anthracycline chemotherapy.

Nottingham grade of cancer		2^nd^ line drugs received (this is given after the three weeks of completion of anthracycline chemotherapy)	
Low nuclear grade in situ CA	0.5%	None	2.6%
Grade I	15.3%	Docetaxel	65.3%
Grade II	51.5%	Paclitaxel	23.4%
Grade III	32.7%	Nano paclitaxel	8.7%

Type of anthracycline		Blood relatives	
Doxorubicin	80.1%	With cancer	24.0%
Epirubicin	19.9%	Without cancer	76.0%

Number of anthracycline cycles given		Marital states	
04 cycles	98.5%	Married	75.5%
06 cycles	1.5%	Unmarried	24.5%

Patients with		Receptor status	
Diabetes mellitus	42.9%	ER positives	25.5%
Dyslipidemia	35.2%	PR positives	19.4%
Neither	21.9%	Her 2 positives	1.0%
		ER and PR positives	48.0%
		ER and Her 2 positives	3.1%
		PR and Her 2 positives	1.5%
		ER, PR, and Her 2 positives	1.5%

CA: carcinoma; ER: estrogen receptor; PR: progesterone receptor; Her 2: human epidermal growth factor receptor 2.

**Table 3:** Comparison of echocardiographic parameters among the four stages.

Echocardiographic parameters	One day prior to anthracycline chemotherapy (baseline readings, stage I)	One day after 1^st^ dose of anthracycline chemotherapy (stage II)	One day after last dose of anthracycline chemotherapy (stage III)	Six months after completion of anthracycline chemotherapy (stage IV)
EF (%)	68.77 ± 0.77	68.77 ± 0.77	65.58 ± 1.6^∗∗∗^	63.46 ± 2.2^∗∗∗^
FS (%)	31.33 ± 1.1	31.33 ± 1.1	30.66 ± 1.0^∗∗∗^	29.92 ± 0.9^∗∗∗^
PWT, LV (mm)	11.22 ± 0.9	11.22 ± 0.9	10.92 ± 0.7^∗∗∗^	10.53 ± 0.8^∗∗∗^
IVS, LV (mm)	10.22 ± 0.9	10.22 ± 0.9	10.06 ± 0.8^∗^	9.56 ± 0.8^∗∗∗^
LVEDD (mm)	44.61 ± 2.7	44.61 ± 2.7	45.47 ± 2.4^∗∗^	46.63 ± 1.9^∗∗∗^
LVESD (mm)	30.71 ± 2.0	30.71 ± 2.0	31.63 ± 1.9^∗∗∗^	32.49 ± 1.6^∗∗∗^

All values are expressed as mean ± standarddeviation (*n* = 196). *p* values ^∗^ < 0.05, ^∗∗^ < 0.01, ^∗∗∗^ < 0.001 compared to the baseline readings. Paired *t*-test was applied to compare echocardiographic parameters between the baseline and one day and six months after the completion of anthracycline chemotherapy. EF: ejection fraction; FS: fractioning shortening; PWT, LV: posterior wall thickness (left ventricle); IVS, LV: thickness of interventricular septum (left ventricle); LVEDD: left ventricular end-diastolic diameter; LVESD: left ventricular end-systolic diameter.

**Table 4:** Correlation between the basic characteristics and the LVEF six months after the completion of anthracycline chemotherapy.

Variables	Correlation coefficient (*p* value)
Age	-0.141 (0.048)^∗^
Weight	0.595 (0.0001)^∗∗∗^
Height	0.332 (0.0001)^∗∗∗^
BMI	0.487 (0.0001)^∗∗∗^
Cumulative dose	0.087 (0.227)
Radiation	0.292 (0.0001)^∗∗∗^

EF: ejection fraction; BMI: body mass index; *p* values ^∗^ < 0.05, ^∗∗^ < 0.01, ^∗∗∗^ < 0.001 is considered as significant.

**Table 5:** Simple linear and multiple linear regression models of echocardiography parameters six months after the completion of anthracycline chemotherapy.

Echocardiography measurements	Adjusted *R*^2^ (%) for the models	
	Simple linear	Multiple linear
EF	88.8	87.8
FS	41.8	39.7
PWT, LV	41.8	41.4
IVS, LV	22.7	22.7
LVEDD	58.4	57.6
LVESD	55.9	57.4

EF: ejection fraction; FS: fractioning shortening; PWT, LV: posterior wall thickness (left ventricle); IVS, LV: thickness of interventricular septum (left ventricle); LVEDD: left ventricular end-diastolic diameter; LVESD: left ventricular end-systolic diameter.

**Table 6:** Analysis of the risk factors of cancer patients independently associated with cardiotoxicity (logistic regression analysis).

Characteristics	OR	95% CI	*p* value
Age (years)			
50–59	0.60	0.29–1.24	0.167
60–79	1.14	0.53–2.4	0.730
BMI (kg/m^2^)			
Overweight versus normal	4.02	1.97–8.23	0.0001
Obese versus normal	4.21	1.68–10.53	0.002
Nottingham grade of cancer			
Grade II vs. grade I	0.62	0.25–1.49	0.283
Grade III vs. grade II	1.76	0.71–4.36	0.218
Treatment with doxorubicin	1.80	0.80–4.07	0.158
Diabetes mellitus	0.72	0.39–1.33	0.292
Dyslipidemia	1.28	0.69–2.38	0.431
Trastuzumab	2.19	0.73–6.55	0.159
Radiation	1.33	1.08–1.65	0.006

OR: odds ratio; CI: confidence interval; BMI: body mass index. *p* < 0.05 was considered as the level of significance.

## Data Availability

The datasets used and/or analyzed during the current study are available from the corresponding author on reasonable request.
